# Significant Cation Effects in Carbon Dioxide–Ionic Liquid Systems

**DOI:** 10.1002/cphc.201200970

**Published:** 2013-01-02

**Authors:** Oldamur Hollóczki, Zsolt Kelemen, László Könczöl, Dénes Szieberth, László Nyulászi, Annegret Stark, Barbara Kirchner

**Affiliations:** [a] Wilhelm-Ostwald-Institut für Physikalische und Theoretische Chemie, Universität LeipzigLinnéstr. 2, 04103 Leipzig (Germany) E-mail: bkirchner@uni-leipzig.de; [b] Department of Inorganic and Analytical Chemistry, Budapest University of Technology and EconomicsSzt. Gellért tér 4, 1111 Budapest (Hungary) E-mail: nyulaszi@mail.bme.hu; [c] Institut für Technische Chemie, Universität LeipzigLinnéstr. 3–4, 04103 Leipzig (Germany)

**Keywords:** ab initio calculations, carbon dioxide, dispersion interactions, ionic liquids, molecular dynamics

## Abstract

Carbon dioxide–ionic liquid systems are of great current interest, and significant efforts have been made lately to understand the intermolecular interactions in these systems. In general, all the experimental and theoretical studies have concluded so far that the main solute–solvent interaction takes effect through the anion, and the cation has no, or only a secondary role in solvation. In this theoretical approach it is shown that this view is unfounded, and evidence is provided that, similarly to the benzene–CO_2_ system, dispersion interactions are present between the solute and the cation. Therefore, this defines a novel site for tailoring solvents to tune CO_2_ solubility.

## 1. Introduction

Among their numerous potentially advantageous properties,[1–5] ionic liquids (ILs) exhibit unique properties in CO_2_ absorption.[6] Although they dissolve CO_2_ much better than other gases, as shown by Brennecke and co-workers,[7] they are practically insoluble in supercritical CO_2_, which makes them perfect candidates not only for capturing CO_2_ from industrial waste gases,[8,9] but also for gas separations, extraction processes,[7] and bi- or multiphase catalysis involving CO_2_.[6] For the improvement of these applications, an understanding of the solubility of CO_2_ is required through the identification of the CO_2_–IL interaction sites.[10] Accordingly, several experimental studies were performed to compare the Henry’s law constants for different ILs,[6,8,11–13] and based on the observed trends a picture of CO_2_ solvation in ILs was established, which could be justified by the corresponding theoretical investigations.[6,11–14] The general wisdom of these studies is that while the anion plays a crucial role in the solute–solvent interplay, the cation–CO_2_ interaction is rather limited to small contributions from the side chain,[12] and so far no significant direct effect of the cationic head group has been reported. Accordingly, the formation of a hydrogen-bond-like[15–17] interaction in imidazolium-based ILs between the CO_2_ oxygen atoms and the cationic ring hydrogen atoms was excluded, since neither the Henry’s law constants in the experiments changed, nor were any discrepancies noticed in the microscopic structure of the solvent in classical molecular dynamics (MD) simulations through the exchange of the most acidic (thus, most likely interacting) H2 atom by a methyl group,[11] thus inferring a certain unimportance of the cation.

**Figure 1:**
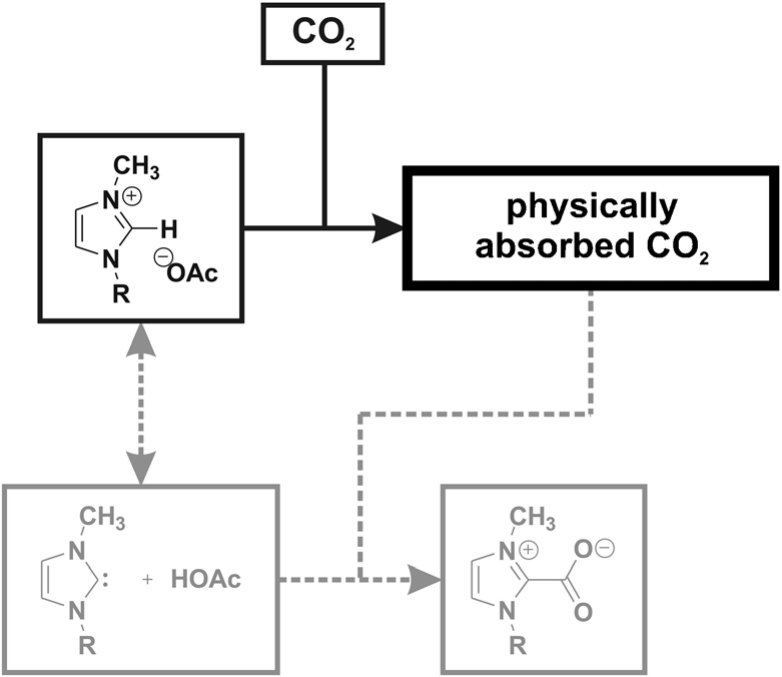
Mechanistic picture of CO_2_–[C_*n*_C_1_Im][OAc] systems (Im=imidazolium). The system investigated herein is highlighted by a thicker frame.

The anion–CO_2_ interaction can be described as a Lewis acid–base reaction, and accordingly, by the increasing basicity of the anion, this interaction becomes stronger.[10] Interestingly, in the presence of basic anions the formation of carbenes may also occur by proton transfer from the cation to the anion,[18–21] and since carbenes are known to react with CO_2_ yielding imidazolium carbonates,[22] in the case of sufficiently basic anions the formation of such structures is expected. In agreement, the chemical absorption of CO_2_ in 1,3-dialkylimidazolium acetates has been suggested based on the significantly increased solubility of CO_2_ in these ILs,[23] and Rogers and co-workers[24] (and later several other groups)[25–28] recently revealed the formation of 1,3-imidazolium carboxylates in the same system. According to the above information on IL–CO_2_ systems and carbene formation, it is reasonable to assume the mechanism depicted in Figure 1: physical absorption of CO_2_ in the 1,3-dialkylimidazolium acetate, followed by reaction of the solute with the carbene that is accessible in these ILs. However, to improve and to exploit this reaction more effectively, a more detailed mechanistic insight is required for each step of the process.

In this theoretical study we investigate the initial step, the physical absorption of CO_2_ in 1-ethyl-3-methylimidazolium acetate ([C_2_C_1_Im][OAc]), as the first of a series of investigations on this apparently interesting but rather complex system (Figure 1). Moreover, due to the higher basicity of the acetate anion, increased anion–CO_2_ interactions are expected.[10] Therefore, in this system the role of the cation in the solvation of the CO_2_ should be even lower, which allows a careful view in revisiting the presence of cation–CO_2_ interactions in imidazolium-based ILs in general.

## Computational Methods

Ab initio molecular dynamics (AIMD) simulations[29–31] were carried out with periodic boundary conditions, which—in contrast to classical MD simulations based on a force field—allow the monitoring of unforeseen changes in the electronic structure. Given that the bending of the CO_2_ is of high importance in the anion–CO_2_ interaction[32] (note that CO_2_ is usually kept linear in force fields),[12] there is a need for the description of the electronic structure in extreme molecular interactions, and thus the advantage of AIMD is clearly indicated.

The simulated system was built by inserting a single CO_2_ molecule into the simulation box, which was obtained in a series of previous simulations by our group on the neat IL, and successfully reproduced many of its experimental physical properties.[33] The resulting system of 36 ion pairs and one CO_2_ molecule was equilibrated for 5 ps in an NVT ensemble employing a massive Nosé–Hoover thermostat, and then simulated for 68 ps at 350 K in an NVT ensemble by applying a regular Nosé–Hoover thermostat, by the CP2k program package,[34] and by using the BLYP-D functional, the MOLOPT-DZVP-SR-GTH basis sets, and GTH pseudopotentials. The applied functional—in significant difference to previous AIMD studies on IL–CO_2_ systems[35,36]—also includes Grimme’s most recent dispersion correction (D3),[37,38] which is essential in IL systems.[37,39–41] The analysis of the trajectories was performed with TRAVIS.[42]

Static quantum chemical calculations were carried out by applying the BLYP-D/def2-TZVPP, BLYP/def2-TZVPP, and (RI)MP2/def2-TZVPP methods and basis sets by the TURBOMOLE 6.0[43] (applying increased convergence criteria on the optimization of 10^−4^ a.u., and on the SCF of 10^−8^ hartree) and SNF[44] program packages, and M06-2X, B97-D, B3LYP, and MPW1K DFT with the 6-311+G** basis set by the Gaussian 09 program package.[45]

## 2. Results and Discussion

**Figure 2:**
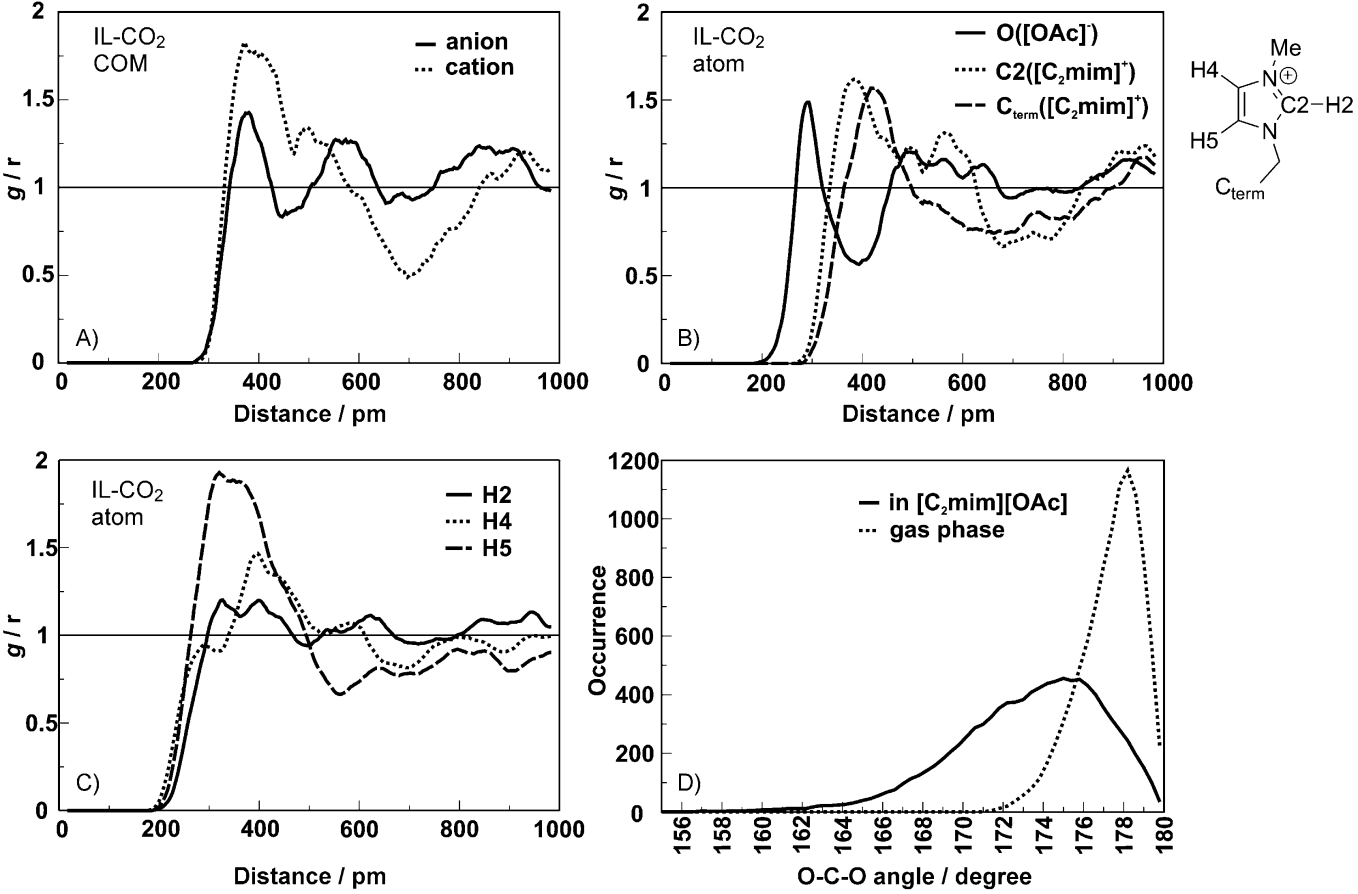
Radial distribution functions, *g*(*r*), between centers of mass (COMs) (A), measured from the C atom (B) and from the O atom (C) of the CO_2_, and the angular distribution of CO_2_ in the gaseous phase and in the IL (D). mim=methylimidazolium, term=terminal.

**Figure 3:**
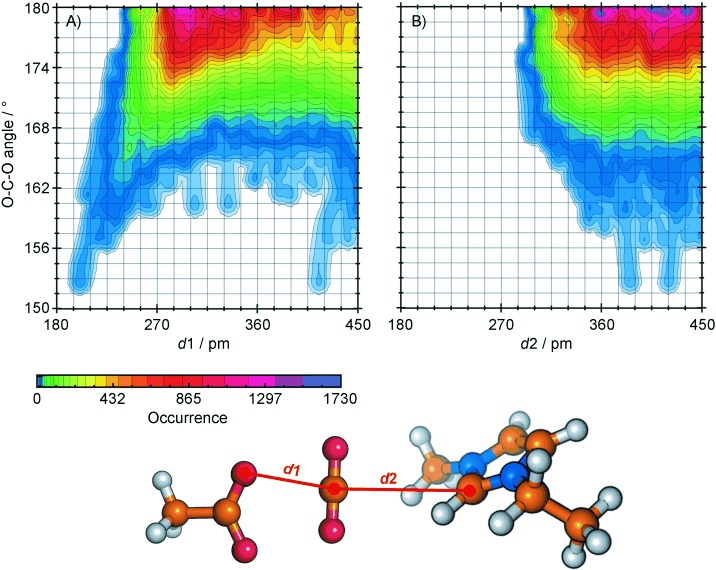
Combined distribution function showing the CO_2_ bond angle against the depicted distances.

On the basis of the radial distribution functions (RDFs), the acetate oxygen–CO_2_ carbon distances are the shortest (2–300 pm), providing a very pronounced peak (black line in Figure 2 B) similar to that found before in other ILs.[11,14] However, our results show noticeable deviations compared to a previous AIMD study on the same IL containing 50 mol % CO_2_.[35] Here, the C(CO_2_)–O([OAc]^−^) distances are longer (black line in Figure 2 B) and also the CO_2_ bond angles are larger, although the bending is still more pronounced than that in the gas phase (Figure 2 D). These differences may originate from the different molar ratios (1:1[35] vs. 1:36), the different simulation temperature (298[35] vs. 350 K), or the much shorter simulation time (12[35] vs. 68 ps) and the lack of proper account for dispersion interaction in the previous AIMD study.[35] In full agreement, by static calculations on isolated acetate–CO_2_ assemblies lacking dispersion correction we observed, for example, the shortening of the distances between the aforementioned two atoms (by ca. 10 pm, see the Supporting Information), which clearly affects the outcome of the AIMD simulations as well. Nevertheless, despite these differences, the entries in the lower left part of the combined distribution function (CDF) in Figure 3 A clearly indicate that whenever the anion’s oxygen atom is close to the CO_2_’s carbon atom, the bending of the CO_2_ is increased, which—together with the observed short anion–CO_2_ distances—points to the importance of the anion–CO_2_ interactions.

**Figure 4:**
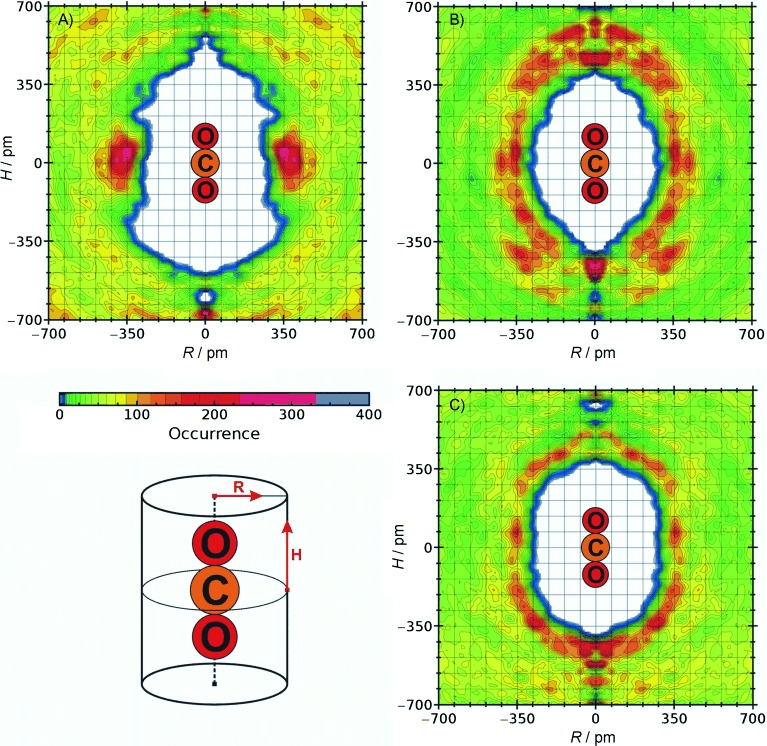
Spatial distribution of the anionic (A) and the cationic COMs (B), and the terminal carbon atom of the cationic ethyl group (C) around the CO_2_.

Surprisingly, the cationic centers of mass (COMs) are at similar distances to the solute as the anionic ones (Figure 2 A), while the corresponding peak is higher, thus showing that the cation also contributes to the solvent–solute interactions by providing more neighbors (ca. five versus the ca. one anion). Interestingly, although such pronounced peaks have previously been observed in cation–CO_2_ pair correlation functions, they were related to “packing effects” rather than to solute–solvent interactions. However, by comparing the spatial distribution functions (SDFs) of the two ions, a different viewpoint can be obtained (Figure 4). The interaction with the anion is clearly directed to the CO_2_’s carbon atom; thus, the acetate ions are located mainly in a thin specific ring around the solute. The cations can be observed in a similarly structured manner around the CO_2_, but these regions of interaction cover its whole surface; thus, a picture of a cation cage emerges (Figure 4 B). This high local structuring of the ions around the solute is in contrast to the picture that CO_2_ solely occupies already existing voids in the IL.[6,11]

Given that the approach of the acetate anion toward the solute polarizes the CO_2_ by bending it into a negatively charged carboxylate group, one may infer that this bending strengthens the interaction with the cations, as was found in an analogous reaction between amines and CO_2_ in imidazolium-based ILs.[46] Surprisingly, the CDF in Figure 3 B clearly shows that the closer the solute is to the cation, the less bent it is, as for the lower C(CO_2_)–C2 distances there are no entries corresponding to lower O–C–O angles of the solute. Thus, instead of cooperation, *competition* is indicated between the anion and the cation for interacting with the CO_2_. The finding that despite this competition the aforementioned cation cage is formed clearly shows the significance and strength of the cation–CO_2_ interactions.

Although there is a large peak in the RDF between the H5 and the CO_2_’s oxygen atoms, the large (above 200 pm) distances between any ring hydrogen atoms and the solute oxygen (Figure 2 C) support the previous findings[11] in pointing to the lack of hydrogen bonding with CO_2_ in such systems. These substantial distances in the H2 RDF (black line in Figure 2 C), together with the lack of any significant peaks in it, also perfectly explain why the methylation at position 2 has no effect on the CO_2_ solubility.[11] Similarly to Costa Gomes and co-workers,[12] a pronounced side-chain CO_2_ peak was obtained (dashed line in Figure 2 B), which suggests that this moiety also has some impact. However, the SDF of the terminal side-chain carbon around the solute exhibits significantly less structuring than that of the cationic COM (Figure 4 C), whereas the C2([C_2_mim]^+^)–C(CO_2_) distances (dotted line in Figure 2 B) show that the CO_2_ molecule is, in fact, similarly close to the cationic ring.

**Figure 5:**
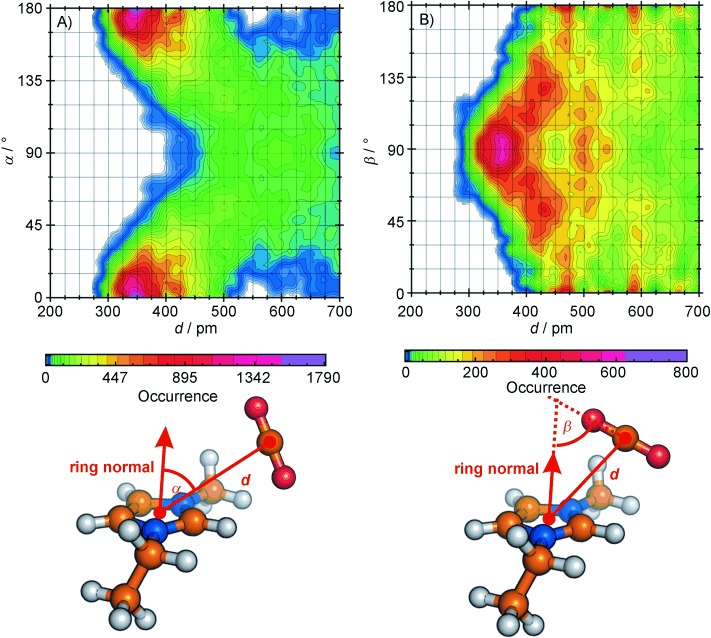
Combined distribution functions representing the orientation of the CO_2_ with respect to the cationic ring, based on the depicted geometrical measures.

Furthermore, according to the CDFs shown in Figure 5, the CO_2_ is strictly above the ring of the nearby cations, and oriented mostly in a parallel fashion to the ring plane, although perpendicular conformers can also be observed. This on-top arrangement of the CO_2_ around the nearby imidazolium cations has been observed before,[47] and was related to the competition between the anion and the solute for interacting with the H2 atom. Clearly, this competition has an influence; however, we would like to point out that these findings also indicate the presence of a dispersion interaction with the cationic π system, which is analogous to that in the benzene–CO_2_[48] and pyridine–CO_2_[49] systems. The similar ring–CO_2_ distances (328.6 pm for benzene at the MP2/aug-cc-pVTZ level,[48] and ca. 360 pm in the present simulation) are also noteworthy. As mentioned above, the interaction with the cation is apparently enhanced by the linearity of the CO_2_; thus, the lack of a proper dispersion description in the simulations may result in the overestimation of the CO_2_’s bending. Although this picture provides a possible explanation for the deviations from the previous study,[35] it should also be kept in mind that the different molar ratios may alter the number of available interacting cations.

**Figure 6:**
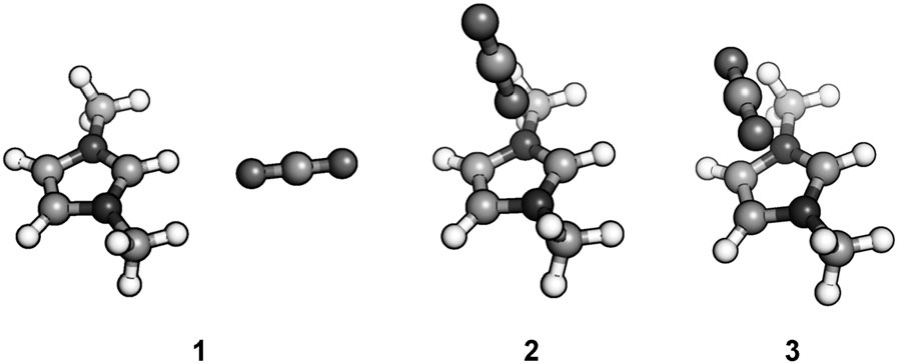
Obtained structures for the system composed of a 1,3-dimethylimidazolium cation and a CO_2_ molecule.

To further analyze the interaction between carbon dioxide and the imidazolium π system, static quantum chemical calculations were carried out by a number of different theoretical methods (see Computational Methods) on the CO_2_–1,3-dimethylimidazolium cation model system. The geometry of the obtained three minima (Figure 6) further stresses the analogy with the aforementioned benzene–CO_2_ interplay.[48,50] The most stable minimum (**1**) possesses the CO_2_ molecule in the ring plane, apparently in interaction with the H2 atom. The lack of this structure in the present AIMD trajectory, and also in the previous MD simulations, is due to the competition between the anion and the solute for this position (cf. with the neat IL).[33] The two other structures (**2** and **3**) are about 3 and 6 kJ mol^−1^ less stable, with the CO_2_ positioned approximately 320 pm above the cationic ring in either a perpendicular (**2**) or a parallel (**3**) fashion. The Bader analysis[51] of both **2** and **3** supports the presence of an interaction between the CO_2_ and the cationic π system, by exhibiting unprecedented bond critical points between the cation’s nitrogen atoms and the CO_2_’s oxygen atoms. The bond critical points between the methyl hydrogen atoms and the solute oxygen atoms allow concluding interactions with the methyl groups of the cation. The relative energies are comparable in all methods applied, but the importance of the dispersion’s proper treatment was again observed, as during the geometry optimizations by the BLYP and B3LYP functionals either the rearrangement of **2**-like and **3**-like structures to **1** was observed, or the CO_2_–cation distance increased to 1300 pm (for more data, see the Supporting Information). Although the cation–CO_2_ interaction energies are somewhat lower than those for the anion–CO_2_, the cationic cage around the solute suggested by the AIMD calculations makes it necessary to consider the effect of these π interactions.

## 3. Conclusions

In this theoretical study the interactions between CO_2_ and imidazolium-based IL cations have been investigated by AIMD simulations and static quantum chemical calculations, on the one hand to provide insight into the first step of CO_2_ absorption in 1,3-dialkylimidazolium acetates, and on the other hand to revisit those results in the literature in which the main solute–solvent interaction in IL–CO_2_ systems in general takes effect through the anion.

Undeniably, there is a strong anion effect and a moderate side-chain effect on CO_2_ solvation in ILs, as was proposed previously by experimental (Henry’s law constants) and theoretical (classical MD) studies. However, even in the case of such a strong anion–CO_2_ interplay as that with the acetate anion, the occurrence of an attractive interaction between the cationic π system and the solute has been evidenced in the study reported herein. Although nonaromatic cations may form other kinds of interactions as well,[52] and the corresponding interaction energies may therefore be similar, our results, and the fact that imidazolium-based ILs dissolve more CO_2_ than pyrrolidinium ones,[13] indicate that boosting the CO_2_–aromatic interactions may indeed increase CO_2_ solubility in ILs. This knowledge may allow not only a deeper understanding of the solubility of CO_2_ in imidazolium-based ILs, but also may provide novel perspectives in tailoring[52] of ILs by incorporating aromatic units into the ions, for example, by using aromatic anions or aryl-functionalized side chains. Such modification may allow the improvement of nonreactive CO_2_ capture processes, and may also open paths to the development of ILs that are soluble in supercritical CO_2_.

## Supplementary material

Detailed facts of importance to specialist readers are published as ”Supporting Information”. Such documents are peer-reviewed, but not copy-edited or typeset. They are made available as submitted by the authors.
