# Supplementary material for: Significant Cation Effects in Carbon Dioxide–Ionic Liquid Systems
Source: Chemphyschem. 2013 Jan 2;14(2):315–20. doi: 10.1002/cphc.201200970 (PMC3597992; doi:10.1002/cphc.201200970)
Supplement: Supplementary file 1 [file cphc0014-0315-SD1.pdf]

CHEMPHYSCHEM

Supporting Information

© Copyright Wiley-VCH Verlag GmbH & Co. KGaA, 69451 Weinheim, 2013

Significant Cation Effects in Carbon Dioxide–Ionic Liquid Systems

Oldamur Hollóczki,[a] Zsolt Kelemen,[b] László Könczöl,[b] Dénes Szieberth,[b]
László Nyulászi,*[b] Annegret Stark,[c] and Barbara Kirchner*[a]

cphc_201200970_sm_miscellaneous_information.pdf

Supporting Information

Table of contents

Isomers of 1,3-dimethylimidazolium cation - CO_2 structure, and their relative energies 3
Difference in the anion – CO2 interactions with BLYP and BLYP-D methods with the def2-TZVPP basis set 4
XYZ geometries and total energies of the 1,3-dimethylimidazolium cation at different levels of theory 5
XYZ geometries and total energies of CO_2 at different levels of theory 7
XYZ geometries and total energies of the acetate anion at different levels of theory 8
XYZ geometries and total energies of the acetate - CO_2 structures at different levels of theory 9
XYZ geometries and total energies of the 1,3-dimethylimidazolium cation - CO_2 structures at the B97-D/6-311+G** level 10
XYZ geometries and total energies of the 1,3-dimethylimidazolium cation - CO_2 structures at the BLYP-D/def2-TZVPP level (optimized with scfconv=8 and GCART=4) 12
XYZ geometries and total energies of the 1,3-dimethylimidazolium cation - CO_2 structures at the BLYP/def2-TZVPP level (optimized with scfconv=8 and GCART=4) 14
XYZ geometries and total energies of the 1,3-dimethylimidazolium cation - CO_2 structures at the M06-2X/6-311+G** level 15
XYZ geometries and total energies of the 1,3-dimethylimidazolium cation - CO_2 structures at the B3LYP/6-311+G** level 17
XYZ geometries and total energies of the 1,3-dimethylimidazolium cation - CO_2 structures at the RI-MP2/def2-TZVPP level 18
XYZ geometries and total energies of the 1,3-dimethylimidazolium cation - CO_2 structures at the MPW1K/6-311+G** level 20
Color version of Figures 3 and 5 21

Isomers of 1,3-dimethylimidazolium cation - CO_2 structure, and their relative energies

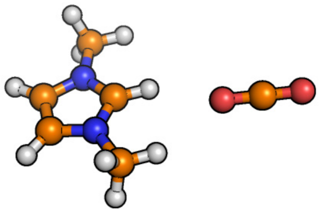

1

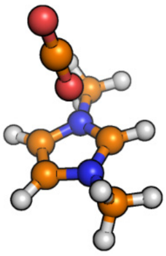

2

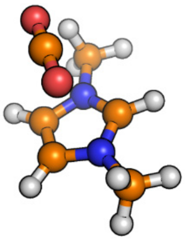

3

	B97-D/6-311+G** [a]	BLYP-D/def2-TZVPP [b]	BLYP/def2-TZVPP [b]	M06-2X/6-311+G** [a]	B3LYP/6-311+G** [a]	RI-MP2/def2-TZVPP [b]	MPW1K/6-311+G** [a]
1	0.0	0.0	0.0	0.0	0.0	0.0	0.0
2	3.0	3.3	-	1.7	-	2.0	5.7[d]
3	6.5	6.6	-	4.7	15.1[c]	6.3	-

[a] calculated with the Gaussian 09 program package

[b] calculated with the TURBOMOLE 6.0 program package

[c] the distance of CO_2 from the cationic ring in the optimized structure is ca. 1300 pm, indicating no interaction at the given level

[d] a rearrangement during optimization results in a structure, which has the CO_2 apparently in interaction with one of the rear hydrogen atoms (H4), and the methyl group

Difference in the anion – CO_2 interactions with BLYP and BLYP-D methods with the def2-TZVPP basis set

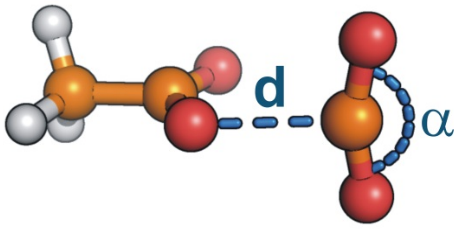

	BLYP/def2-TZVPP	BLYP-D/def2-TZVPP
**d**	316.7 pm	326.3 pm
α	156.7°	159.4°

XYZ geometries and total energies of the 1,3-dimethylimidazolium cation at different levels of theory

B97-D/6-311+G**

E(B97-D/6-311+G**)=-305.090096

N	0.000701	0.030329	-0.000342
C	0.000875	0.009709	1.342761
N	1.273592	0.030010	1.771865
C	2.111902	0.064635	0.669274
C	1.313282	0.064839	-0.442620
C	1.710457	0.017584	3.182616
C	-1.196662	0.018438	-0.864867
H	-0.876943	-0.018159	1.973251
H	3.188301	0.085601	0.770308
H	1.561300	0.086024	-1.494913
H	0.825247	-0.010577	3.824249
H	2.325070	-0.871517	3.358795
H	2.287759	0.925648	3.386161
H	-1.205298	0.926963	-1.476260
H	-1.167238	-0.870176	-1.504228
H	-2.087383	-0.010317	-0.230933

BLYP-D/def2-TZVPP

E(BLYP-D/def2-TZVPP)=-305.228614

N	0.103488	-0.003740	-1.092874
C	0.888465	-0.005781	-0.000014
N	0.103480	-0.003298	1.092826
C	-1.223504	0.000733	0.684936
C	-1.223394	0.000194	-0.684844
C	0.573590	0.005093	2.494824
C	0.573520	0.005465	-2.494852
H	1.662925	-0.089389	-2.501055
H	0.124752	-0.839374	-3.027031
H	0.279238	0.948048	-2.968589
H	1.662600	-0.094390	2.501063
H	0.283442	0.949472	2.967467
H	0.121286	-0.837122	3.028154
H	-2.039275	0.000577	1.394414
H	-2.039138	-0.001888	-1.394357
H	1.969562	-0.006103	0.000003

M06-2X/6-311+G**

E(M06-2X/6-311+G**)=-305.165816

N	0.004951	0.030389	0.007245
C	0.007508	0.010018	1.337997
N	1.267761	0.029995	1.765413
C	2.101816	0.064368	0.669834
C	1.309528	0.064619	-0.433241
C	1.703225	0.017624	3.166710
C	-1.183900	0.018510	-0.852932
H	-0.868174	-0.018047	1.966953
H	3.174297	0.085411	0.769241
H	1.557836	0.085921	-1.481301
H	0.823606	-0.010429	3.805674
H	2.312739	-0.866725	3.343543
H	2.275518	0.920758	3.370874
H	-1.194375	0.921781	-1.460258
H	-1.157049	-0.865707	-1.487195
H	-2.070325	-0.009451	-0.223439

XYZ geometries and total energies of CO_2 at different levels of theory

B97-D/6-311+G**

E(B97-D/6-311+G**)=-188.5378278

C	0.000000	0.000000	0.000000
O	0.000000	0.000000	1.168947
O	0.000000	0.000000	-1.168947

BLYP-D/def2-TZVPP

E(BLYP-D/def2-TZVPP)=-188.654968

C	0.000000	0.000000	0.000000
O	0.000000	0.000000	-1.173090
O	0.000000	0.000000	1.173090

M06-2X/6-311+G**

E(M06-2X/6-311+G**)=-188.574881

C	0.000000	0.000000	0.000000
O	0.000000	0.000000	1.155094
O	0.000000	0.000000	-1.155094

XYZ geometries and total energies of the acetate anion at different levels of theory

B97-D/6-311+G**

E(B97-D/6-311+G**)=-228.456570

C	0.015773	-0.019983	-0.015546
C	0.030794	-0.007388	1.565629
O	1.176270	0.019057	2.095470
O	-1.105103	0.005695	2.117732
H	-0.893645	-0.507082	-0.402088
H	0.017903	1.025011	-0.372058
H	0.913894	-0.515312	-0.418138

BLYP-D/def2-TZVPP

E(BLYP-D/def2-TZVPP)=-228.571216

C	0.014325	0.000000	-0.030778
C	0.007359	0.000000	1.556220
O	1.142002	0.000000	2.116024
O	-1.146537	0.000000	2.076531
H	1.037526	0.000000	-0.436217
H	-0.527337	-0.886313	-0.400389
H	-0.527337	0.886313	-0.400389

M06-2X/6-311+G**

E(M06-2X/6-311+G**)=-228.500643

C	0.015791	-0.020063	-0.008313
C	0.030543	-0.007873	1.549826
O	1.163583	0.017742	2.075364
O	-1.092408	0.006325	2.097617
H	-0.887771	-0.503626	-0.385275
H	0.017196	1.017959	-0.357146
H	0.908951	-0.510466	-0.401074

XYZ geometries and total energies of the acetate - CO_2 structures at different levels of theory

BLYP-D/def2-TZVPP

E(BLYP-D/def2-TZVPP)=-417.246006

C	1.430361	0.019644	-0.534203
C	-0.097614	-0.037906	-0.194493
O	-0.883976	0.180663	-1.181121
C	-3.010677	0.058550	-0.416958
O	-3.200360	1.197295	-0.139492
O	-0.399961	-0.278896	0.999950
O	-3.235967	-1.091262	-0.608711
H	1.608749	0.114548	-1.614362
H	1.876587	0.882508	-0.015265
H	1.924489	-0.885380	-0.149770

BLYP/def2-TZVPP

E(BLYP-D/def2-TZVPP)=-417.239008

C	1.418447	-0.000431	-0.579255
C	-0.083624	-0.056581	-0.153686
O	-0.917685	0.109323	-1.116784
C	-2.994889	0.051279	-0.505276
O	-3.210278	1.200221	-0.278849
O	-0.327148	-0.244026	1.060585
O	-3.254698	-1.100807	-0.657314
H	1.537340	0.041971	-1.668662
H	1.884501	0.889615	-0.132671
H	1.945749	-0.878397	-0.181970

XYZ geometries and total energies of the 1,3-dimethylimidazolium cation - CO_2 structures at the B97-D/6-311+G level**

1

E(B97-D/6-311+G**)=-493.634574

C	-0.005164	0.030355	-0.022740
N	-0.038382	-0.003070	1.362382
C	1.223413	-0.020869	1.823545
N	2.061165	0.000205	0.774094
C	1.313039	0.032414	-0.391769
C	-1.259089	-0.017212	2.191828
C	3.535898	-0.008735	0.851433
O	3.321719	-0.100888	4.483068
C	3.867545	-0.131399	5.524142
O	4.407389	-0.161543	6.552343
H	-0.966662	-0.042463	3.245353
H	-1.840389	0.888744	1.989903
H	-1.847643	-0.908272	1.948768
H	3.827475	-0.040998	1.904240
H	3.914477	-0.894189	0.329769
H	3.922880	0.902193	0.382387
H	1.776656	0.053724	-1.368389
H	-0.908806	0.049325	-0.616250
H	1.521671	-0.047494	2.862216

2

E(B97-D/6-311+G**)=-493.633418

C	-0.047947	0.176944	0.053859
C	-0.036886	0.271076	1.419358
N	1.280006	0.123144	1.824956
C	2.045806	-0.056422	0.736942
N	1.261322	-0.023068	-0.350409
C	1.759091	0.162069	3.220087
C	1.707690	-0.235929	-1.739778
O	1.675610	-3.149454	0.103403
C	1.659625	-4.324154	0.135506
O	1.642604	-5.486319	0.168587
H	2.840405	-0.001763	3.224582
H	1.531022	1.142439	3.651866
H	1.262085	-0.629246	3.791204
H	2.800334	-0.199311	-1.767378
H	1.359503	-1.216527	-2.080422
H	1.295833	0.559172	-2.369424
H	-0.861843	0.237806	-0.655248
H	-0.838104	0.433999	2.126834
H	3.114677	-0.215221	0.738070

3

E(B97-D/6-311+G**)=-493.6321093

C	0.000000	0.000000	0.000000
N	0.000000	0.000000	1.343307
C	1.312219	0.000000	1.785478
C	2.111117	0.002034	0.673079
N	1.272941	0.003033	-0.429138
C	-1.195910	-0.053289	2.207964
C	1.710625	-0.045592	-1.838697
O	0.269112	-3.268838	1.613739
C	0.954323	-3.284626	0.667058
O	1.633456	-3.267220	-0.283955
H	0.837875	0.095990	-2.482506
H	2.432705	0.758387	-2.013219
H	2.166599	-1.022108	-2.033839
H	-2.084743	0.091674	1.587279
H	-1.235357	-1.032346	2.697370
H	-1.130735	0.746795	2.952171
H	1.559458	-0.001411	2.838099
H	3.187563	0.002490	0.571174
H	-0.878247	-0.000874	-0.630690

XYZ geometries and total energies of the 1,3-dimethylimidazolium cation - CO_2 structures at the BLYP-D/def2-TZVPP level (optimized with scfconv=8 and GCART=4)

1

E(BLYP-D/def2-TZVPP)=-493.890538

C	-0.107587	-0.005572	-3.041169
N	-0.960632	0.000163	-1.945412
C	-0.219687	0.004062	-0.821807
N	1.080447	0.001075	-1.168059
C	1.172137	-0.004948	-2.553541
C	-2.437264	0.000358	-2.004535
C	2.221140	0.004469	-0.226855
O	-0.045219	0.002819	2.491383
C	-0.204620	0.000687	3.660756
O	-0.361291	-0.001446	4.815640
H	-2.829117	0.013278	-0.983798
H	-2.776020	0.891601	-2.543180
H	-2.777572	-0.903233	-2.521244
H	1.828712	0.006176	0.792970
H	2.826349	-0.892262	-0.395453
H	2.824419	0.901790	-0.399305
H	2.125586	-0.008111	-3.063232
H	-0.480559	-0.009481	-4.055754
H	-0.597246	0.008711	0.191238

2

E(BLYP-D/def2-TZVPP)=-493.889284

C	-1.216368	0.674762	-1.071517
N	-1.173458	0.708994	0.315813
C	0.113912	0.692152	0.705298
N	0.893175	0.654720	-0.388590
C	0.079968	0.637397	-1.511607
C	-2.346153	0.764747	1.212880
C	2.364945	0.539295	-0.390623
C	0.738529	-3.516670	0.133097
O	0.758700	-2.338034	0.090154
O	0.715874	-4.682142	0.175752
H	-1.995707	0.777984	2.248564
H	-2.918296	1.674558	1.003515
H	-2.970222	-0.118728	1.043691
H	2.746028	0.857845	0.583627
H	2.639470	-0.503987	-0.578180
H	2.768706	1.187888	-1.173758
H	0.489002	0.602686	-2.511438
H	-2.150858	0.687586	-1.614749
H	0.462744	0.698949	1.728075

3

E(BLYP-D/def2-TZVPP)=-493.888022

C	-0.040125	-1.052227	-0.876171
N	1.053622	-1.057744	-0.092381
C	0.648901	-0.979831	1.232185
C	-0.720463	-0.928498	1.232633
N	-1.130725	-0.975962	-0.091650
C	2.454914	-1.070915	-0.562706
C	-2.529031	-0.883743	-0.561660
O	1.258867	2.135811	-0.028186
C	0.087840	2.200705	-0.029081
O	-1.084670	2.227627	-0.034294
H	-2.556951	-1.113368	-1.630471
H	-3.137022	-1.608565	-0.011678
H	-2.894867	0.133790	-0.386527
H	2.464710	-1.294141	-1.633133
H	2.898516	-0.085993	-0.380540
H	3.004974	-1.844577	-0.018297
H	1.360196	-0.969173	2.046154
H	-1.428407	-0.864845	2.047106
H	-0.042313	-1.101099	-1.956161

XYZ geometries and total energies of the 1,3-dimethylimidazolium cation - CO_2 structures at the BLYP/def2-TZVPP level (optimized with scfconv=8 and GCART=4)

1

E(BLYP/def2-TZVPP)=-493.873316

C	-0.107708	-0.005689	-3.059677
N	-0.955039	-0.000072	-1.957908
C	-0.203510	0.003873	-0.839867
N	1.095474	0.001071	-1.194920
C	1.174635	-0.004922	-2.582248
C	-2.433042	0.000326	-2.007528
C	2.244465	0.004500	-0.262923
O	-0.155515	0.003760	2.592949
C	-0.261212	0.000934	3.767898
O	-0.363966	-0.001894	4.929179
H	-2.822321	0.012247	-0.987532
H	-2.778481	0.890237	-2.541212
H	-2.780040	-0.900759	-2.521117
H	1.866167	0.006615	0.760732
H	2.849365	-0.890664	-0.431413
H	2.847765	0.899931	-0.435795
H	2.121243	-0.008010	-3.103494
H	-0.482576	-0.009661	-4.073096
H	-0.573729	0.008312	0.176617

XYZ geometries and total energies of the 1,3-dimethylimidazolium cation - CO_2 structures at the M06-2X/6-311+G level**

1

E(M06-2X/6-311+G**)=-493.748060

C	0.017223	-0.044728	0.011113
N	-0.011442	0.006669	1.387589
C	1.240545	0.048138	1.838588
N	2.069169	0.025200	0.797614
C	1.323760	-0.033357	-0.359139
C	-1.222927	0.015192	2.213597
C	3.533887	0.057663	0.872275
O	3.231468	-0.104058	4.371595
C	3.775949	-0.229483	5.392760
O	4.312991	-0.354155	6.397735
H	-0.931079	0.070632	3.259813
H	-1.825837	0.884166	1.955960
H	-1.784000	-0.901085	2.038338
H	3.821490	0.115505	1.919442
H	3.934754	-0.851751	0.428079
H	3.898518	0.933160	0.337959
H	1.782464	-0.061364	-1.333473
H	-0.884170	-0.084838	-0.577324
H	1.544429	0.088325	2.873800

2

E(M06-2X/6-311+G**)=-493.747428

C	0.005066	-0.036178	0.011244
C	0.006265	-0.014825	1.369264
N	1.323802	0.013407	1.769505
C	2.095127	0.007303	0.686153
N	1.320080	-0.014577	-0.393162
C	1.791834	0.052379	3.157863
C	1.786172	-0.125331	-1.777371
O	1.497428	-2.847703	0.311212
C	1.375936	-4.001044	0.398048
O	1.253589	-5.138906	0.485329
H	2.878959	0.019622	3.161352
H	1.451711	0.973455	3.628142
H	1.399526	-0.811619	3.691116
H	2.838864	0.145701	-1.817097
H	1.653327	-1.151780	-2.116666
H	1.212687	0.558965	-2.399267
H	-0.807950	-0.061484	-0.694755
H	-0.804936	-0.009337	2.077856
H	3.173345	0.011189	0.683830

3

E(M06-2X/6-311+G**)=-493.746281

C	0.021600	-0.004576	0.000664
N	-0.046088	0.129622	1.368389
C	1.192288	0.195274	1.850069
N	2.052946	0.112049	0.836971
C	1.339024	-0.012399	-0.334897
C	-1.275524	0.080474	2.167004
C	3.517299	0.141646	0.938870
C	1.401742	-2.961583	0.927054
O	0.341494	-2.894096	1.382707
O	2.464501	-2.990583	0.476744
H	-1.047701	0.400453	3.181384
H	-2.008230	0.755307	1.729375
H	-1.650253	-0.942469	2.173956
H	3.791923	0.243728	1.986569
H	3.914863	-0.791481	0.542490
H	3.897647	0.992086	0.375843
H	1.826106	-0.092547	-1.292747
H	-0.863248	-0.085721	-0.608759
H	1.456427	0.297794	2.891182

XYZ geometries and total energies of the 1,3-dimethylimidazolium cation - CO_2 structures at the B3LYP/6-311+G level**

1

E(B3LYP/6-311+G**)=-493.960268

N	0.195175	-0.045184	-0.213318
C	0.075805	-0.064930	1.117923
N	1.297359	-0.023121	1.658812
C	2.228783	0.024450	0.639005
C	1.538844	0.010887	-0.533459
C	1.596360	-0.029194	3.098915
C	-0.915658	-0.077555	-1.176139
H	-1.855119	-0.114328	-0.628640
H	-0.889303	0.821489	-1.791003
H	-0.826491	-0.963422	-1.804141
H	0.658668	-0.064497	3.648240
H	2.195830	-0.906282	3.340639
H	2.139308	0.878622	3.359654
H	3.287648	0.064077	0.830671
H	1.885746	0.036385	-1.552565
H	-0.848340	-0.107172	1.672096
C	-3.036919	-0.299309	4.314419
O	-3.927013	-0.373128	5.043932
O	-2.134932	-0.224498	3.575154

3

E(B3LYP/6-311+G**)=-493.954516

N	0.021178	1.831405	-0.197835
C	0.164012	1.378962	1.051774
N	1.418309	1.606543	1.453755
C	2.101095	2.226376	0.424429
C	1.227019	2.367006	-0.608851
C	1.978554	1.260268	2.769754
C	-1.209308	1.768542	-1.002544
H	-1.994457	1.301874	-0.411313
H	-1.513704	2.777806	-1.277566
H	-1.026940	1.174517	-1.897464
H	1.208778	0.772254	3.363987
H	2.819736	0.580752	2.637337
H	2.305705	2.168682	3.274491
H	3.136034	2.510665	0.512505
H	1.359499	2.796738	-1.587356
H	-0.606714	0.908115	1.639439
C	0.114187	-11.554720	2.396609
O	0.333469	-11.862010	1.299072
O	-0.105371	-11.242488	3.492973

XYZ geometries and total energies of the 1,3-dimethylimidazolium cation - CO_2 structures at the RI-MP2/def2-TZVPP level

1

E(RI-MP2/def2-TZVPP)=-492.887908

C	-0.201306	-0.003762	-3.013596
N	-0.979992	0.001741	-1.878648
C	-0.178780	0.003660	-0.808133
N	1.090668	0.000074	-1.226982
C	1.102373	-0.004674	-2.603321
C	-2.447767	0.001263	-1.846026
C	2.273204	0.003784	-0.355801
O	-0.034055	0.000482	2.479644
C	-0.189156	-0.000908	3.643108
O	-0.341688	-0.002290	4.792154
H	-2.769149	0.028230	-0.809983
H	-2.815510	0.879463	-2.367923
H	-2.814981	-0.902848	-2.322178
H	1.936668	-0.004658	0.675481
H	2.866671	-0.882250	-0.559988
H	2.854340	0.900292	-0.549679
H	2.017387	-0.008084	-3.167529
H	-0.628333	-0.006461	-4.000144
H	-0.493693	0.007385	0.220606

2

E(RI-MP2/def2-TZVPP)=-492.887159

C	-0.525582	1.551189	-1.212918
N	-0.935844	1.574132	0.100317
C	0.106958	1.268887	0.878389
N	1.172864	1.057646	0.102664
C	0.801359	1.223642	-1.210732
C	-2.291050	1.885965	0.569843
C	2.496393	0.632159	0.570551
C	-0.259060	-2.897849	-0.000494
O	0.027549	-1.766094	0.117458
O	-0.543942	-4.016353	-0.118492
H	-2.304285	1.824325	1.653131
H	-2.555318	2.891256	0.255945
H	-2.986720	1.164458	0.152243
H	2.545445	0.770437	1.645779
H	2.638617	-0.415734	0.323999
H	3.253231	1.243832	0.089574
H	1.496780	1.101028	-2.021170
H	-1.194695	1.772311	-2.024685
H	0.089329	1.200383	1.951316

3

E(RI-MP2/def2-TZVPP)=-492.885522

C	-0.243427	-1.065009	-0.862716
N	0.814858	-1.284173	-0.075381
C	0.440616	-1.077102	1.231768
C	-0.882143	-0.728659	1.219604
N	-1.286146	-0.728178	-0.095442
C	2.168024	-1.623720	-0.535590
C	-2.627032	-0.372296	-0.579207
O	1.625731	1.834651	-0.075795
C	0.512882	2.184016	-0.030901
O	-0.609911	2.497746	0.012658
H	-2.687656	-0.611530	-1.636005
H	-3.363344	-0.951184	-0.030546
H	-2.786439	0.691429	-0.428278
H	2.111169	-1.944549	-1.570770
H	2.801816	-0.745654	-0.450713
H	2.551797	-2.433654	0.076539
H	1.129078	-1.198372	2.048651
H	-1.555353	-0.490919	2.023541
H	-0.256013	-1.151354	-1.934880

XYZ geometries and total energies of the 1,3-dimethylimidazolium cation - CO_2 structures at the MPW1K/6-311+G level**

1

E(MPW1K/6-311+G**)=-493.7858484

C	2.554029	-1.042738	-0.005012
C	2.895739	0.262703	-0.005618
N	1.730711	0.981841	0.000176
C	0.714457	0.135860	0.004212
N	1.186364	-1.098900	0.001234
C	1.629646	2.431454	0.002360
C	0.383781	-2.310803	0.003083
O	-2.565910	0.037473	0.005358
C	-3.711465	0.191843	-0.000670
O	-4.841201	0.343765	-0.006669
H	0.583486	2.710827	0.004366
H	2.108716	2.827631	0.890563
H	2.105830	2.829958	-0.886338
H	-0.663114	-2.034799	0.012182
H	0.598119	-2.888306	-0.889086
H	0.611880	-2.894507	0.887746
H	3.163179	-1.925155	-0.008449
H	3.859025	0.733625	-0.009608
H	-0.326876	0.400319	0.009114

2

E(MPW1K/6-311+G**)=-493.7836585

N	-0.799356	1.039793	0.105346
C	-1.768180	0.560001	-0.654094
N	-2.236823	-0.548492	-0.107043
C	-1.540260	-0.788033	1.046932
C	-0.638820	0.207677	1.178733
C	-3.300093	-1.380006	-0.645928
C	-0.032780	2.248618	-0.149641
O	2.539149	-0.051771	-0.047601
C	3.592851	-0.507963	-0.168414
O	4.633225	-0.963427	-0.286691
H	-0.335312	2.662071	-1.103740
H	-0.226594	2.972662	0.633814
H	1.020848	1.997846	-0.180584
H	-3.730310	-0.887330	-1.509092
H	-2.899992	-2.342791	-0.943542
H	-4.068369	-1.513813	0.106821
H	-1.742783	-1.636498	1.670421
H	0.097034	0.390625	1.936208
H	-2.116573	0.997946	-1.569604

Color version of Figures 3 and 5

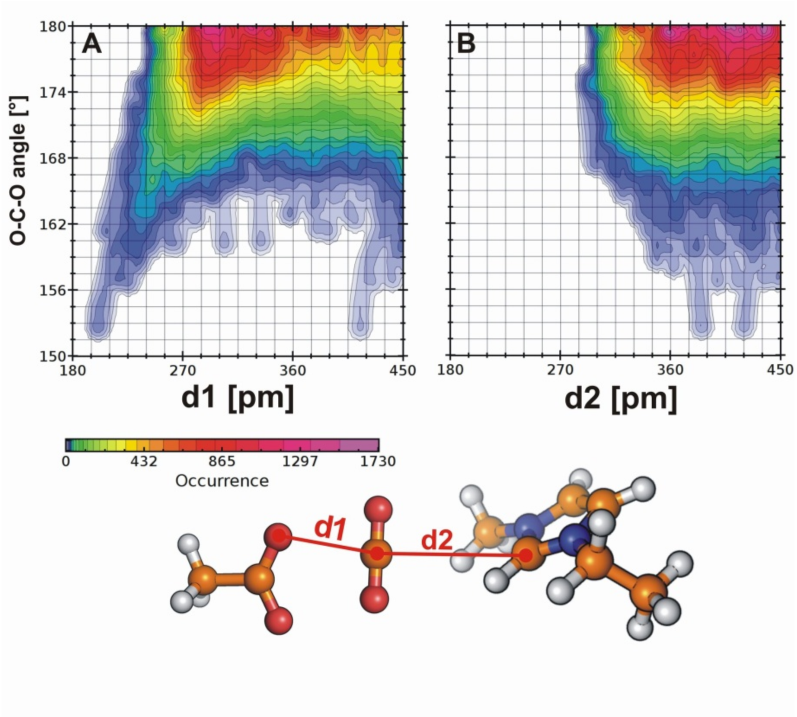

Figure 3. Combined distribution function showing the CO2 bond angle against the depicted distances.

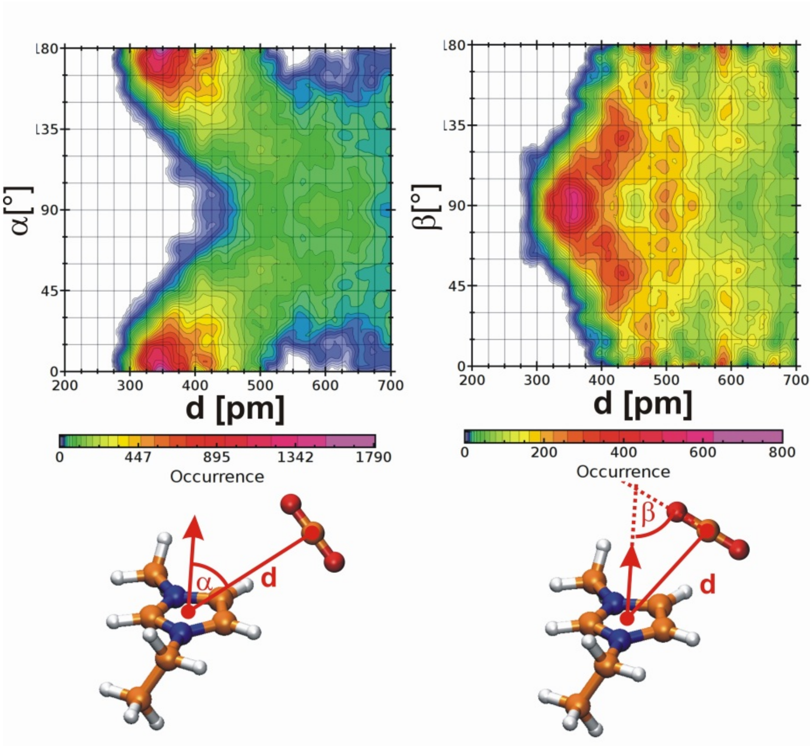

Figure 5. Combined distribution functions, representing the orientation of the carbon dioxide with respect to the cationic ring, based on the depicted geometrical measures.
